# Longitudinal changes of resting-state networks in Parkinson‘s disease

**DOI:** 10.1016/j.nicl.2025.103833

**Published:** 2025-06-25

**Authors:** Matthias Sure, Rasha Hyder, Levent Kandemir, Jan Vesper, Alfons Schnitzler, Esther Florin

**Affiliations:** a Institute of Clinical Neuroscience and Medical Psychology, Medical Faculty, Heinrich Heine University Düsseldorf, Germany; b Bielefeld University, Medical School and University Medical Center OWL, Klinikum Lippe, University Institute for Laboratory Medicine, Microbiology and Clinical Pathobiochemistry, Detmold, Germany; c Cardiff University Brain Research Imaging Centre (CUBRIC), School of Psychology, Cardiff University, United Kingdom; d Department of Functional Neurosurgery and Stereotaxy, Medical Faculty, University Hospital Düsseldorf, Germany; e Department of Neurology, Center for Movement Disorders and Neuromodulation, Medical Faculty, University Hospital Düsseldorf, Germany

**Keywords:** Resting-state network, PAC, MEG, PD, Stun effect

## Abstract

Deep brain stimulation (DBS), but also the sole implantation of the electrodes and dopaminergic medication, can reduce symptoms in Parkinson’s disease (PD) patients. Furthermore, an effect on network activity is known for all three options separately. However, long-term effects have rarely been investigated. Therefore, in the present study, we focus on the long-term impact of dopaminergic medication on whole-brain network activity following DBS electrode implantation.

Therefore, we extracted resting state networks (RSNs) of 20 PD patients (4 females, (59.00 ± 9.72 years) from magnetoencephalography data. We recorded 30 min of resting-state activity two days before and one year after implantation of the electrodes with and without dopaminergic medication, but DBS was turned off. RSNs were obtained based on the phase-amplitude coupling between a low-frequency phase and a high gamma amplitude and examined for differences between conditions (i.e., pre- vs. post-surgery).

We identified three RSNs across all conditions: sensory-motor, visual, and frontal. Each RSN was selectively altered due to a year of disease progression – while patients being treated with dopaminergic medication and DBS. In line with previous literature, we focus on longitudinal changes in RSNs over time after electrode implantation, acknowledging that chronic DBS treatment and other factors may confound the interpretation of these changes. In addition, the alterations found were RSN specific, as dopaminergic medication showed a greater impact on the frontal RSN, and the longitudinal factor expressed by the disease progression was more severe in alterations in the SMN and the visual RSN.

## Highlights

• Three RSNs (sensory-motor, visual, and frontal) are altered by disease progression.
• Less pronounced RSNs changes a year after implantation compared to healthy controls.
• RSN-specific alterations by dopaminergic medication and disease progression.

## 1 Introduction

Parkinson's disease (PD) is a neurodegenerative disorder with a range of symptoms that also occur at rest, exemplified by resting tremor. Related to this, alterations in resting state networks (RSN) compared to healthy controls have been described (de Schipper et al., 2018, Göttlich et al., 2013). These RSNs also change over time, making them potentially a good neural indicator of disease severity (Boon et al., 2020a, Li et al., 2020, Olde Dubbelink et al., 2014, Olde Dubbelink et al., 2013). There is emerging evidence that RSN alterations in PD patients may reflect the effects of clinical treatments, as some studies have reported RSN changes following dopaminergic medication (Schneider et al., 2020), DBS (Kandemir et al., 2023, Oswal et al., 2016), and after electrode implantation (Sure et al., 2023). However, the extent and specificity of these effects require further investigation**.**

Nevertheless, each RSN has been attributed to a certain function, but at the same time, RNSs can be determined simultaneously in a hierarchically organized manner from one dataset (Cordes et al., 2002, Doucet et al., 2011, Smith et al., 2009, Yeo et al., 2011). Therefore, an RSN-based method is suitable to investigate the influence of a disease and its treatment on functional brain networks. Several approaches to determining RSNs based on amplitude correlation or phase information exist (Brookes et al., 2011, Colclough et al., 2016, Marzetti et al., 2014). Nevertheless, for the specific case of RSN in PD patients, phase-amplitude coupling (PAC; Florin and Baillet, 2015) is a suitable choice because PAC values correlated positively with symptom severity in PD and are reduced with treatment (De Hemptinne et al., 2015, De Hemptinne et al., 2013, Tanaka et al., 2022).

In a previous study (Sure et al., 2023), we demonstrated that RSNs undergo immediate changes following DBS electrode implantation, which were attributed to acute surgical effects. However, the long-term impact of DBS implantation and the role of dopaminergic medication in this context remained unclear. In the present study, we aim to investigate how RSNs evolve one year after implantation and how dopaminergic medication modulates these networks over time.

We address this issue by comparing RSN of 20 PD patients before electrode implantation and after chronic dopaminergic and DBS therapy. Measurements were always performed with DBS turned off, but on each recording day, with medication OFF and ON. By comparing resting-state recordings obtained at two time points at least six months apart, both in the medication OFF state, we aimed to capture longitudinal changes in RSNs that are likely to reflect the combined effects of disease progression and chronic DBS therapy. Furthermore, due to the medication ON recordings of the same day, we were able to analyse whether the acute effect of dopaminergic medication on RSNs changes over time.

For both comparisons, we expect alterations in the functional connectivity of PD patients, which will likely differ from longitudinal studies without intermediate DBS (Olde Dubbelink et al., 2014). Furthermore, we expect that due to the longer period after surgery, the stun effect does not affect the functional network changes (Sure et al., 2023). By providing a long-term perspective, this study extends our previous findings and offers novel insights into functional network changes in PD patients.

## 2 Methods

### 2.1 Patients

For this study, brain activity was measured in 20 Parkinson's disease patients with implanted DBS systems (Abbot Infinity with St. Jude Medical Directional lead 6172 (Abbott Laboratories, Lake Bluff, IL, USA)) who were selected for DBS treatment in the subthalamic nucleus according to the guidelines of the German Neurological Society. They all had a positive motor response to levodopa and did not have a diagnosed Parkinson's plus syndrome. They were not selectively chosen for a Parkinson's phenotype, but patients with severe head tremor were excluded as this affects the quality of MEG measurement. All patients gave written informed consent before the start of the measurement. The study was approved by the local ethics committee (study no. 5608R) and conducted in accordance with the Declaration of Helsinki.

MEG was recorded just before electrode implantation (first recording, 2.50 ± 30.89 days) and about one year later (second recording, 445.75 ± 152.44 days). On each of the two measurement days, the Beck Depression Inventory (BDI-II) and the Mini-Mental Status Examination (MMSE; Folstein et al., 1975) were assessed. In addition, the Unified Parkinson's Disease Rating Scale (UPDRS) motor score was evaluated both in the medication OFF state (PD medication was discontinued at least 12 h before) and in the medication ON state (after administration of one and a half times the morning levodopa dose as fast-acting levodopa). In subsequent sections, we have used the akinetic-rigid subscore (The sum of the items 3.3a-c and 3.4–3.8) and refer to it by akinetic-rigid UPDRS. DBS was switched off at least 30 min before recording. Handedness was evaluated using the Edinburgh Handedness Inventory (Oldfield, 1971).

### 2.2 MEG recording

Patients were seated in a MEG in a magnetically shielded room on two days for the first and second recording. DBS was switched OFF at least 30 min prior to the start of the MEG recording and remained off for the duration of the session. On each recording day, three ten-minute blocks were performed in the medication ON and OFF state (cumulating into 60 min of recording time per recording day). The OFF recording was completed first, and the ON recording was performed at least 30 min after administration of the fast-dissolving medication. However, for three patients, the ON and OFF measurements were performed on two different days, with the patients taking their normal prescribed medication for the ON state. The patients were asked not to move during the recording and to fixate with their eyes on a black cross on white paper. An eye tracker (iView X 2.2, SensoMotoric Instruments, Teltow, Germany) was used to check whether the eyes were open. In addition to the patient recordings, 5 min of empty room MEG data were recorded on each measurement day.

All recordings were performed with a sampling frequency of 2400 Hz and a low-pass filter of 800 Hz. An electrocardiogram and an electrooculogram were measured to detect artifacts due to heartbeats and eye movements. To allow later source reconstruction, four head position indicator coils were taped to the head and digitized via the Polhemus system (Polhemus Isotrack, Colchester, VT, USA) for co-registration with individual T1-weighted anatomical MRIs (depending on scanner availability: 3 T Trio Tim, 3 T Prisma, 1.5 T Avanto, 1.5 T Avanto-fit, 1.5 T Sola; all with 1 mm3 voxel size). In addition, another 100 points were distributed over the skull, and the three anatomical landmarks, Nasion, RPA, and LPA, were digitized.

### 2.3 Signal processing

For data processing and analysis of the MEG data, Matlab (version R 2016b; The MathWorks, Inc., Natick, MA, USA), customized scripts, and the Matlab-based toolbox Brainstorm (https://neuroimage.usc.edu/brainstorm/Introduction; Tadel et al., 2011) were used.

Identification of artifacts was carried out independently of each other by two persons. If consensus was not reached for a time segment, that time segment was considered to contain artifacts and was excluded from further analysis. A standard step in artifact removal was the initial application of the MNE-Python (Gramfort et al., 2013) implementation of tSSS (Taulu and Hari, 2009) through Brainstorm with a default subspace correlation limit of 0.98. No prominent artefacts associated with the implanted DBS system or its leads were observed, as the materials used were specifically optimized to minimize magnetic interference. In addition, common artifacts that follow the same pattern, such as eye blinks or cardiac artifacts, were removed using custom-made SSPs. Time periods with irregular artifacts, such as motion artifacts, were removed from all channels. Of the 20 patients considered, after artifact cleaning, 18 could be further analyzed from the first-OFF, 18 from the first-ON, 17 from the second-OFF, and 19 from the second-ON measurement. Notably, the patients excluded in each session varied, so we were unable to perform paired tests. Furthermore, line noise of 50 Hz and its harmonics up to 600 Hz were removed from all channels with a notch filter with a 3-dB bandwidth of 1 Hz.

Cleaned data was projected to the source space. For this purpose, the MEG and MRI data were coregistered using the Polhemus data. Furthermore, cortical surfaces were extracted with Freesurfer (Dale et al., 1999) from the individual T1-MRIs and down-sampled to 15,002 sources within Brainstorm. For the forward solution, the Brainstorm implementation of overlapping spheres (Huang et al., 1999) was chosen, and the inverse problem was solved using the Brainstorm implementation of weighted minimum-norm imaging. The noise covariance matrix required for this was determined using the daily empty room measurements, which were pre-processed in the same way as the actual recording.

### 2.4 Resting-state network estimation

**Fig. 1:**
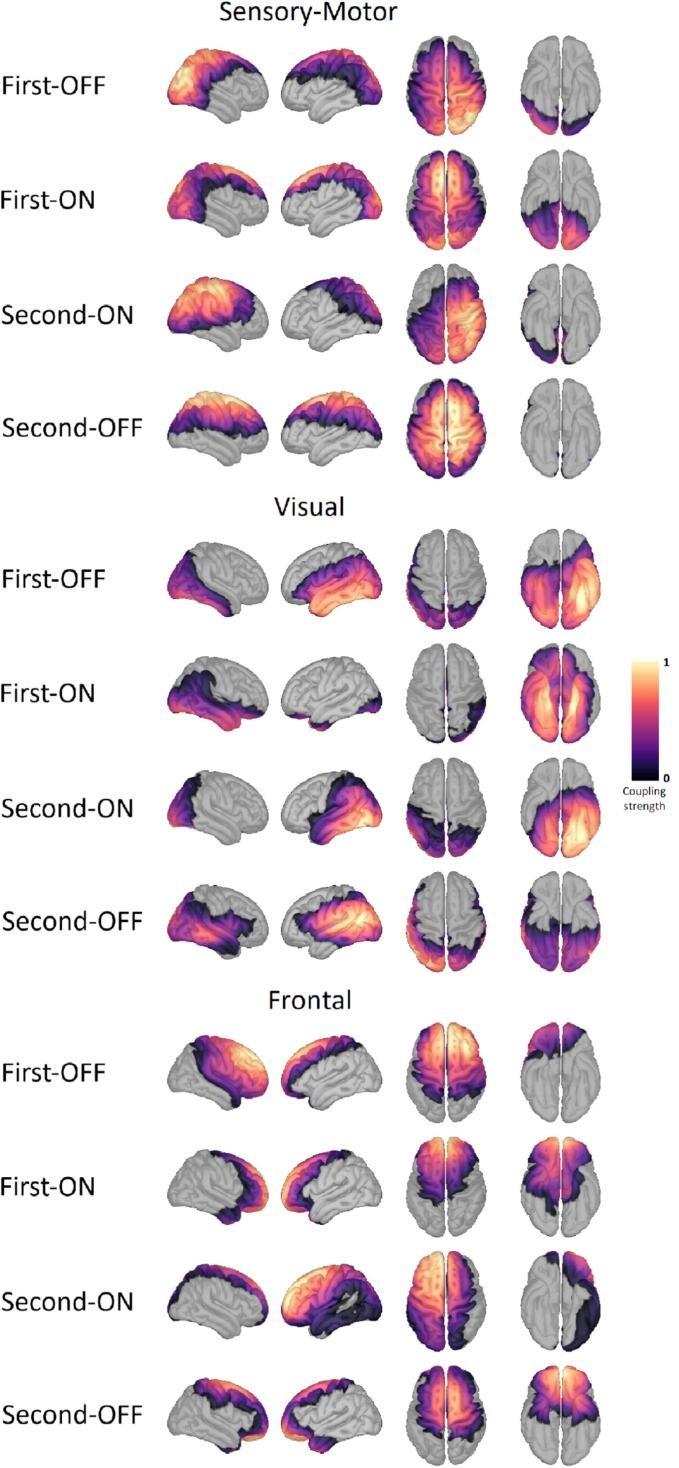
Resting state networks investigated in four different Parkinson’s disease conditions The resting state networks (RSN) under consideration (sensory-motor, visual, and frontal) are arranged in blocks one below the other. The rows of each block display the network for the recordings before electrode implantation (first) OFF and ON medication, and the recordings after (second) electrode implantation ON and OFF medication are below. The color scale marks the coupling strength from 0 to 1, with a warmer color indicating a higher coupling strength. No threshold was applied. It can be seen that the focus of the coupling strength for the individual RSNs is located in different regions of the brain. When comparing the conditions, it is also noticeable that the topography of the RSN changes.

The data-driven megPAC method was used to determine RSN, which has the maximum phase-amplitude coupling (PAC; Özkurt and Schnitzler) as its central parameter: For each reconstructed source, the pair of frequencies is determined with the maximum coupling $\left({\hat{f}}_{\varphi },{\hat{f}}_{a}\right)$ between the phase of a low frequency $f_{\varphi }$ ∈ [2, 30] and the amplitude of a high frequency$f_{a}$ ∈ [80, 150]. Subsequently, a new time series, called megPAC, is generated. For this purpose, the maxima and minima of the low-frequency component are determined, and the signal of the high-frequency component is interpolated in between. For comparability with fMRI-RSN methods, this signal was down-sampled to 10 Hz. The source reconstructed data based on individual patient anatomy were projected onto the standard brain ICBM152 via FreeSurfer's spherical registration. On the standard brain, the data were spatially smoothed with a Gaussian filter with a full width at half maximum of 7 mm. The time series prepared in this way were concatenated from all patients. To extract group-level resting-state networks, the correlation between all cortical time series was calculated, resulting in a large correlation matrix. For computational tractability and to minimize the influence of uneven MEG sensitivity, a dimensionality reduction step is performed by using a subset of evenly distributed sources. Based on this, the correlation between all time series was calculated, and ten cortical maps − the RSN − with coupling strength between 0 and 1 were determined as the principal spatial modes via singular value decomposition. In accordance with conventions in fMRI resting-state analysis (Smith et al., 2009) and the original megPAC study (Florin and Baillet, 2015), we extracted the first ten RSN components. Of the ten possible RSNs, the sensory-motor network (SMN; see Fig. 1 top), the visual RSN (see Fig. 1 center), and the frontal RSN (see Fig. 1 bottom) were further investigated, as they were reliably identified across all four conditions and corresponded topographically to known RSN, as validated in healthy control data (Mertiens et al., 2023). For more detailed information on the megPAC method, the reader is referred to Florin and Baillet (https://github.com/FlorinNeuro/MEEG-restingstate/tree/main/PAC_megPAC. 2015).

### 2.5 Statistical testing between RSNs from the different conditions

To compare the coupling strength of the RSN between conditions, the jackknife procedure with leave one out (Stute, 1996), as described in more detail in Sure et al. (2023), was used. Here, RSNs are calculated repeatedly for a condition, omitting one patient at a time. Thus, N sets of RSNs were generated for a condition with N patients.

To determine the investigated RSNs from the individual jackknife runs, RSNs were first determined for all patients in a condition. The SMN, the frontal RSN, and the visual RSN were determined and used as template RSN. The Phi coefficient (Yule, 1912) was determined between the template RSNs and the jackknife RSNs. A cutoff value was drawn at 40 % of the maximum coupling strength while determining the Phi coefficient. The RSNs from the jackknife runs with the highest coefficient compared to the template RSN were further evaluated as SMN, visual RSN, and frontal RSN, respectively.

A 2-factor ANOVA was calculated with the factors medication (ON, OFF) and recording day (first, second) to test for statistical differences in the coupling strength of the RSN between conditions. The ANOVA was calculated separately for each vertex. For an RSN, those vertices were included in the ANOVA, which had 40 % of the maximum coupling strength in at least one of the four conditions. As a post-hoc test, a two-tailed unequal variance *t*-test was calculated. For multiple comparisons, we corrected using the false discovery rate.

### 2.6 Overlap with healthy RSN

To compare the RSNs against a constant external variable, the spatial correspondence of the RSNs considered here was compared with the RSNs in healthy controls, which were determined by Sure et al. (2023) and Mertiens et al. (2023). Here, the Phi coefficient between the RSNs in healthy controls and the RSNs determined in each jackknife run was determined. Before determining the Phi coefficients, a minimum threshold of 40 % was imposed on the coupling strength of the RSN. The Phi coefficients thus determined the differences between the four tested conditions using a two-tailed *t*-test followed by Bonferroni correction.

### 2.7 Analysis of the low-frequency component

As changes in dopamine concentration can lead to changes in beta oscillations (Iskhakova et al., 2021), we examined the low-frequency component of the PAC. We determined the frequency whose phase maximally coupled to high gamma for each vertex. These frequency values were determined based on the subject’s anatomy and projected onto the standard brain for comparability. The median frequency was determined over the vertices that belonged to an RSN so that each RSN and condition pair had a value. We calculated a 3-factor (RSN, medication, recording session) ANOVA with Bonferroni correction for the subsequent post-hoc analysis using the median frequencies.

## 3 Results

Data from 20 patients (59.00 ± 9.72 years), four of whom were female, were included in the results. MMSE, BDI, and akinetic-rigid UPDRS distributions were not normally distributed according to the Kolmogorov-Smirnov test. The Wilcoxon signed-rank test revealed that there were no significant differences for both the MMSE (first: 28.89 ± 1.47; second: 28.81 ± 0.83; p = 0.35) and the BDI (first: 10.89 ± 7.78; second: 12.93 ± 10.27; p = 0.16). However, the akinetic-rigid UPDRS was significantly higher OFF medication compared to ON in the first recording (OFF: 20.53 ± 7.02; ON 11.89 ± 5.53; p = 0.0006), as well as in the second recording (OFF: 20.85 ± 8.20; ON 18.35 ± 7.57; p = 0.0438). The akinetic-rigid UPDRS values between first and second recording differed significantly ON medication (p = 0.0175) but not OFF medication. However, between the first measurement and the second measurement, DBS was used as an additional treatment option for the patients, which significantly reduced the morning dose of levodopa (first: 173.68 ± 58.02 mg; second: 117.65 ± 87.44 mg; p = 0.0189).

The comparison of the coupling strength of the RSN revealed significant differences between all three RSNs, both for the main effects recording session (first vs. second) and medication status (ON, OFF) and the subsequent post-hoc tests. These results are considered separately for each RSN.

### 3.1 Sensory-motor network

**Fig. 2:**
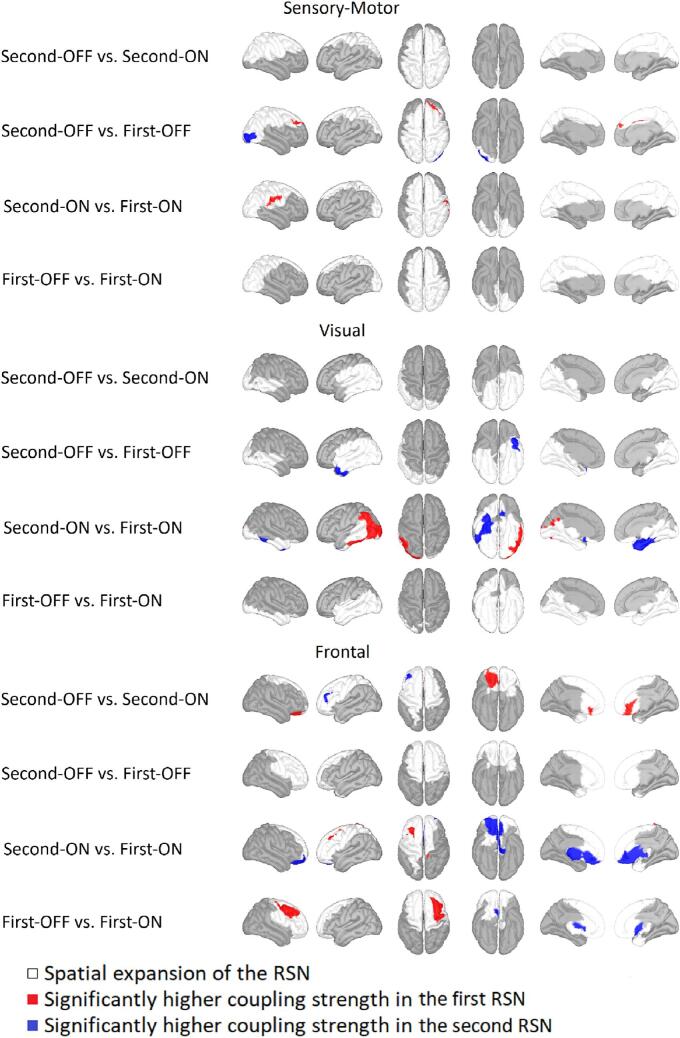
Alteration in the resting state networks The alterations in the resting state networks (RSN) under consideration (sensory-motor, visual, and frontal) are displayed in blocks one below the other. The RSNs were compared after recordings from Parkinson’s patients after electrode implantation (second) and before (first) and with medication (ON) and without (OFF). Areas belonging to either one and/or both recording conditions are marked in white. Red indicates a significantly higher coupling strength for the former measurement group and blue for the latter one. Significance is given at a p-value below 0.05 after false discovery rate correction for the number of vertices, networks, and conditions based on a two-sided Student’s t-test (HC vs. OFF/ON independent t-test, ON vs. OFF paired t-test). For the sensory-motor RSN in particular, alterations were only present in the comparisons second vs. first recording but not OFF vs. ON. Also, for the visual RSN, alterations were only present in the comparisons second vs. first recording but not OFF vs. ON but more severe than for the sensory-motor RSN. However, for the frontal RSN, alterations were more prominent for the OFF vs. ON comparisons. (For interpretation of the references to color in this figure legend, the reader is referred to the web version of this article.)

For the SMN, there were significant main effects for the recording session and medication status. However, post-hoc, the only significant differences were for the recording session (see Fig. 2 top). ON medication, there was an increase in coupling strength for the second recording in the right temporal region of the pre-, post-, and supramarginal gyrus. OFF medication, however, coupling strength increased rostrally in the right frontal lobe and the right cingulate gyrus. In addition, compared to the first recording, in the OFF coupling strength decreased at the right lateral occipital gyrus.

**Fig. 3:**
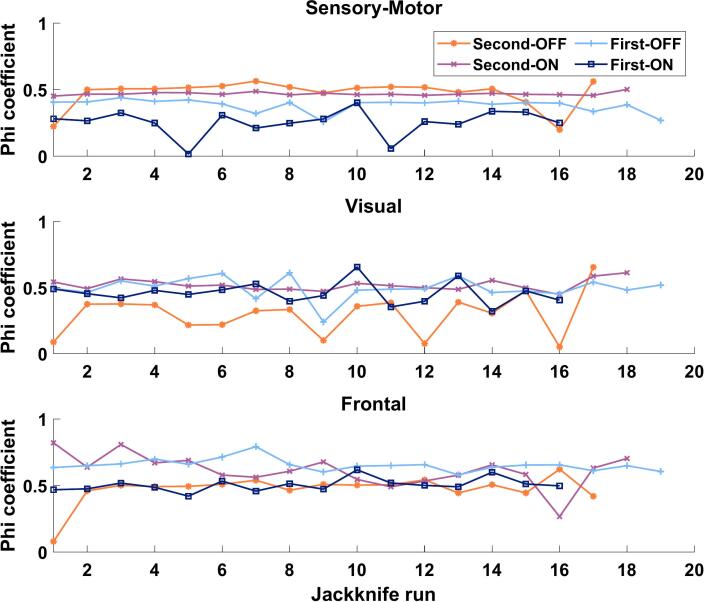
Spatial correspondence between resting-state networks of healthy controls and Parkinson's patients The spatial correspondence between resting-state networks calculated with the phi-coefficient based on the thresholded and then binarized coupling strength between the RSNs of PD conditions (second-OFF, line indicator: asterisk; second-ON, x; first-OFF, +; first-ON, square) and the healthy controls is plotted for each jackknife run (x-axis). The number of patients and, therefore, jackknife runs differed between conditions.

The SMN showed significantly smaller spatial agreement with the HC-RSN in the first ON compared to the other conditions (see Fig. 3 top; second-OFF: 0.473 ± 0.104 [mean ± std], second ON: 0.469 ± 0.012, first-OFF: 0.383 ± 0.050, first-ON: 0.254 ± 0.097; p < 0.002; t > 5.067; df >= 15). In addition, first-OFF RSNs showed a significantly smaller agreement than second ON RSNs (p = 0.000; t = 7.179; df = 17).

### 3.2 Visual network

For the visual RSN, only first- and second-recordings were significantly different in terms of coupling strength but not between medication OFF and ON (see Fig. 2 center). OFF medication, there was a significantly higher coupling strength before electrode implantation in the area of the left temporal pole. ON medication coupling strength for the first recording was significantly higher in the right inferior temporal and fusiform gyrus. At the second recording, there was a higher coupling strength in the left cuneus and cingulate gyrus and the left inferior temporal gyrus, inferior parietal lobule, and lateral occipital sulcus.

Comparing the spatial agreement of visual RSN to that of HC, the second OFF had a significantly lower overlap with HC RSN than for all three other conditions (see Fig. 3 center; second-OFF: 0.301 ± 0.159 [mean ± std], second-ON: 0.520 ± 0.042, first-OFF: 0.497 ± 0.083, first-ON: 0.459 ± 0.084; p < 0.002; t > 5.109; df >= 15). There were no significant differences between the other conditions.

### 3.3 Frontal network

In the second recording, the coupling strength of OFF medication was higher at the right lateral orbitofrontal gyrus in the frontal RSN and lower at the left rostral middle frontal gyrus compared to ON medication (see Fig. 2 bottom). At the first recording, the coupling strength OFF medication was higher than ON medication within the right caudal middle frontal gyrus and right precentral gyrus and lower at the bilateral middle orbitofrontal cortex. ON medication, the coupling strength of the frontal RSN at the left caudal middle frontal gyrus was also higher in the second recording than in the first recording. In contrast, the coupling strength at the bilateral orbitofrontal gyrus was significantly higher in the first recording than in the second recording.

For the frontal RSN, only first-OFF had a significantly higher spatial agreement with HC-RSN than second-OFF and first-ON (see Fig. 3 bottom; second-OFF: 0.473 ± 0.111 [mean ± std], second-ON: 0.614 ± 0.123, first-Med-OFF: 0.654 ± 0.046, first-ON: 0.506 ± 0.049; p < 0.001; t > 6.956; df >= 15).

### 3.4 Low-frequency component

Initially, we investigated whether the low-frequency component relevant to the meg PAC determination differed between conditions. Here, a 3-factor ANOVA with the factors RSN (SMN, Frontal, Visual), medication status (ON, OFF), and recording session (first vs. second) revealed only a significant main effect for the RSNs (F(1,201) = 39.68; p = 1.85e-9; η^2^p = 0.164), but without significant post-hoc test.

## 4 Discussion

In the present work, both dopaminergic medication and a year of disease progression modulate the coupling strength within RSNs. Overall, the RSNs are stronger modulated by a year of disease progression than by dopaminergic medication. Only in the frontal network was a clear effect due to dopaminergic medication. This indicates, that the effect of dopaminergic medication does not change over time in the motor and visual system– even with the implanted DBS electrodes and the reduced dopaminergic medication. The changed RSN without medication likely indicate the further disease progression, even though the UPDRS scores did not change over the course of one year. These findings build upon our previous work (Sure et al., 2023), where we described acute post-surgical effects on RSNs. Compared to the immediate post-operative phase, some RSNs appear to stabilize over time, while others continue to exhibit alterations. This suggests that the initially observed changes are not solely due to surgical effects but also reflect long-term neural adaptations. Furthermore, our findings emphasize the significant role of dopaminergic medication in shaping network dynamics over extended periods.

While our primary analytic unit were the resting-state networks (RSN), we also report differences at the level of specific brain regions within each RSN. This approach allows us to identify which subregions within a network are most affected, providing greater anatomical specificity while preserving the functional network context. Such a combination of network- and region-level analysis is commonly used to interpret RSN alterations in clinical populations (Filippi et al., 2019, Olde Dubbelink et al., 2014).

### 4.1 Medication and disease progression affect PD RSN

PD is characterized mainly by motor symptoms but also by non-motor symptoms. Since the effects of dopaminergic medication and DBS occur not only in motor domains, changes in different RSNs were to be expected. This is also reflected in previous fMRI work (Filippi et al., 2019, Ruppert et al., 2021, Steidel et al., 2022) and was the case for the considered RSNs using the megPAC method (Kandemir et al., 2023, Mertiens et al., 2023, Sure et al., 2023).

In the present case, RSNs were examined longitudinally while patients were treated with DBS and dopaminergic medication during the year between the two measurements. The observed longitudinal changes in RSN coupling strength could result from a combination of disease progression (Olde Dubbelink et al., 2014, Olde Dubbelink et al., 2013), chronic DBS treatment (Hacker et al., 2018, Muehlberg et al., 2023), and ongoing dopaminergic medication (Schneider et al., 2020). As we did not include a matching control group for each factor, the specific contribution of each factor cannot be isolated in the present study. Our findings should therefore be interpreted as describing the overall longitudinal effect of clinical management in this patient group. In any case, the alterations in the coupling strength also depended on whether dopaminergic medication was taken or not. Here, in particular, the considered RSNs were differentially influenced by dopaminergic medication and the year of disease progression. Significant differences for the SMN and the visual RSN in the post-hoc analysis were only present in the comparison first vs. second recording but not OFF vs. ON recording. For the frontal RSN, however, differences were mainly for OFF vs. ON recording but also first vs. second recording ON medication. These differences could be due to DBS – being continuously applied between the two recording dates till 30 min before recording onset − and levodopa differently influencing brain activity (Kandemir et al., 2023, Mueller et al., 2020, Mueller et al., 2018). Since the progression of Parkinson's disease has an influence on brain activity (Huang et al., 2007, Pieperhoff et al., 2022), this could also indicate to what extent the pathological progression of the disease modulates the effect of dopaminergic medication on brain activity.

In terms of symptom improvement, concomitant application of dopaminergic medication and DBS enhances symptom suppression compared to only one treatment alone (Vizcarra et al., 2019). Since there was chronic DBS and medication administration before the second recording, but the ON recording was only with dopaminergic medication but no DBS, this could explain why there was no direct correlation between the clinical scores collected and the RSN and why there were significant differences between first and second recording for the RSN but not for the clinical scores. This could be another indication for the point that electrophysiological activity links particularly to specific clinical symptoms (Boon et al., 2020b) and indicates that broad RSN alterations do not map precisely with complex medical scores (e.q., akinetic-rigid UPDRS, BDI).

Irrespective of this, it is striking that for the SMN and the visual RSN, different alterations between the first and second recording could be determined depending on the medication, but no direct difference between OFF and ON medication, neither in the first nor second recording, could be found. This suggests that the longitudinal factor, be it disease progression or influence of DBS, plays a more critical role in forming these two RSNs than dopaminergic medication.

### 4.2 Influence on specific RSN

For the SMN, OFF medication, the coupling strength shifted toward frontal areas for the first recording compared to the second recording, hence a decrease in coupling strength in occipital areas and an increase frontally. At the same time, ON medication coupling strength increased in somatosensory areas, while no post-hoc differences between OFF and ON occurred. Therefore, dopamine influences longitudinal changes of the SMN in PD. The observed enhancement of coupling strength could indicate a reinforcing effect by joint administration of DBS and dopaminergic medication (Vizcarra et al., 2019). As DBS was turned off during the time of recording, this could reflect neuroanatomical changes induced by chronic DBS (Munro et al., 2023), which challenges the alterations evoked by disease progression (Pieperhoff et al., 2022). Furthermore, the enhanced coupling strength ON medication also fits because motor areas are more excitable and activated after dopamine administration (Burciu and Vaillancourt, 2018; Casarotto et al., 2019). This overrides the shift of the SMN OFF medication.

The alteration between the first and second recordings was more pronounced for the visual RSN than for the SMN. At the edges of the visual RSN, there was an attenuation of the coupling strength and, only ON medication, an enhancement in the visual cortex when comparing the second with the first recordings. Overall, this speaks for increased activity in the visual cortex after electrode implantation with chronic DBS and administration of dopaminergic medication. At the RSN level alterations by dopamine (Michell et al., 2006) and electrode implantation (Sure et al., 2023) have been described previously. Additionally, it is known that DBS, after being turned off, still affects the visual system (Gupta et al., 2022). Generally, individual symptoms reappear within minutes of switching off the DBS, but it can take up to four hours for all symptoms to become fully pronounced (Temperli et al., 2003). It is possible, however, that the effects − medication, stun effect by electrode implantation, and DBS − can only be detected if the influences occur simultaneously.

Comparing the frontal RSN with the literature, it most closely resembles the default mode network (DMN; Brookes et al., 2011, Mantini et al., 2007), for which modulation by dopamine (Delaveau et al., 2010), electrode implantation (Luo et al., 2021) but also DBS (Bai et al., 2022) is known. That the changes for the individual comparisons differ significantly between the conditions could be because, on the one hand, the functional connectivity of the DMN is influenced by DBS (Bai et al., 2022) and the pure electrode implantation (Luo et al., 2021), but on the other hand the general deactivation depends essentially on the dopamine level (Delaveau et al., 2010). The fact that there were no changes only for the second recording vs. the first recording OFF medication suggests that dopamine is the more central variable for the functional connectivity of the DMN, and the presence of an electrode is only a secondary factor. These deviating changes compared to those of the SMN and the visual RSN indicate that the influence of DBS electrode implantation and dopaminergic medication must be considered separately for different functional systems. This is also supported by findings reporting different influences on different RSNs by DBS and dopaminergic medication (Kandemir et al., 2023).

### 4.3 Overlap with HC RSN

When examining the spatial agreement of PD RSN with HC RSN, a higher agreement was expected for the second-ON measurement, because of the positive effect of dopaminergic medication and chronic DBS on PD symptoms. Although the agreement of HC RSN with the PD RSN second-ON did not differ significantly from the other conditions, the second-ON phi-coefficients had higher values. Overall, most of the conditions had comparable phi-coefficients, and there were mostly isolated deviations. The fact that these deviating conditions differed for each RSN (SMN: first-ON, Visual: second-OFF, Frontal: first-OFF) suggests that dopaminergic medication and preceding chronic DBS have different pronounced effects on different RSNs. In contrast, Sure et al. (2023) showed that RSNs from PD patients without DBS implanted have a significantly higher concordance with HC RSN than post-surgery RSN. However, the difference between the present work and that of Sure et al. (2023) is that there is a year between the postoperative measurements, so the different results in the correspondence with HC RSN are likely due to the decreased stun effect. Therefore, the spatial distribution of RSN can be altered by inflammatory processes due to invasive electrode implantation but also, as shown in this work, due to disease progression or chronic DBS and chronic medication.

### 4.4 Influence of the low-frequency component

Since investigating the low-frequency component for the maximum PAC only showed a significant effect for RSN, this suggests that the low-frequency component could be used to distinguish RSN. This also fits the results of Sure et al. (2023), which indicated an effect of electrode implantation, if the measurements were performed directly before and after electrode implantation. As there was no significant effect in the present data for the recording session – directly before and one year after electrode implantation − it suggests that the so-called stun effect affects RSN, but similar to the clinical observations, decreases with increasing time after electrode implantation. This further suggests that chronic DBS, even when temporarily switched off, and dopaminergic medication influence functional networks but do not alter the low-frequency of the estimated PAC.

### 4.5 Limitation

On average, the first- and second-recording are more than one year apart. During this year, the patients were treated with chronic DBS, which can lead to neuroanatomical changes. Additionally, it is also possible that DBS turned off 30 min before the start of the measurement still has a reverberating effect on brain activity. Furthermore, due to the clinical DBS therapy, the prescribed dose of dopaminergic medication was reduced, which led to different dopamine levels in the ON phases of the first and second measurements. However, the continued chronic administration of dopaminergic medication itself can also influence brain activity and, thus also the RSNs examined. It was not possible to correct for either or disentangle those short and long-term DBS and medication dose effects.

Even if the same functional RSNs can be determined using different methods, it should be noted when comparing them that the individual methods are based on different mathematical principles and could therefore have different foci on the local coupling strength. It should also be noted that the pre-processing could influence the results even with same methods. For example, tSSS was used in Kandemir et al. (Kandemir et al., 2023) and in the present work, as PD patients with implanted pulse generators were examined, whereas no tSSS was used in the work of Mertiens et al. (Mertiens et al., 2023) and Sure et al. (Sure et al., 2023), as they examined patients without a pulse generator.

### 4.6 Conclusion

In summary, our study demonstrates that in PD patients treated with chronic DBS and dopaminergic medication, longitudinal changes in RSN coupling strength occur over the course of one year after electrode implantation. The longitudinal factor also expressed by the disease progression, has a greater impact on the SMN and the visual RSN, and dopaminergic medication on the frontal RSN. Although the design does not allow us to disentangle the specific contributions of each treatment, the results suggest that these effects are RSN-specific. Furthermore, considering the results in Sure et al. (2023) and the present results, the influence of the stun effect on the spatial distribution of the RSNs decreases with time.

Funding

This study was funded by the Volkswagen Foundation (Lichtenberg program 89387) and Deutsche Forschungsgemeinschaft (CRC 295, project C01, Project-ID 424778381 – TRR 295). The funders had no influence on the study design, conduct, or evaluation of the results.

## CRediT authorship contribution statement

**Matthias Sure:** Writing – original draft, Visualization, Validation, Software, Investigation, Formal analysis, Data curation. **Rasha Hyder:** Writing – review & editing, Software, Methodology, Formal analysis, Data curation, Conceptualization. **Levent Kandemir:** Software, Formal analysis, Data curation. **Jan Vesper:** Writing – review & editing, Resources. **Alfons Schnitzler:** Writing – review & editing, Resources, Methodology. **Esther Florin:** Writing – review & editing, Validation, Supervision, Project administration, Methodology, Investigation, Funding acquisition, Formal analysis, Data curation, Conceptualization.

## Declaration of competing interest

The authors declare that they have no known competing financial interests or personal relationships that could have appeared to influence the work reported in this paper.

## Data Availability

Because of privacy law, data are shared upon personal request. Inquiries can be sent to the corresponding author.
